# Features of the waterpipe tobacco industry: A qualitative study of the third International Hookah Fair

**DOI:** 10.12688/f1000research.13796.2

**Published:** 2018-10-23

**Authors:** Neil Singh, Mohammed Jawad, Andrea Darzi, Tamara Lotfi, Rima Nakkash, Benjamin Hawkins, Elie A. Akl

**Affiliations:** 1 Brighton and Sussex Medical School, Brighton, UK; 2 Public Health Policy Evaluation Unit, Imperial College London, London, UK; 3 Clinical Research Institute, American University of Beirut, Beirut, Lebanon; 4 Department of Epidemiology and Biostatistics, Faculty of Health Sciences, American University of Beirut, Beirut, Lebanon; 5 Department of Health Promotion and Community Health, American University of Beirut, Beirut, Lebanon; 6 Department of Global Health and Development, London School of Hygiene and Tropical Medicine, London, UK; 7 Department of Internal Medicine, American University of Beirut, Beirut, Lebanon; 8 Department of Clinical Epidemiology and Biostatistics, McMaster University, Hamilton, ON, Canada

**Keywords:** waterpipe, shisha, hookah, tobacco, smoking, industry

## Abstract

**Background: **Little research has been done to uncover the features of the waterpipe tobacco industry, which makes designing effective interventions and policies to counter this growing trend challenging. The objective of this study is to describe the features of the waterpipe industry.

**Methods:** In 2015, we randomly sampled and conducted semi-structured interviews with 20 representatives of waterpipe companies participating in a trade exhibition in Germany. We used an inductive approach to identify emerging themes.

**Results:** We interviewed representatives and four themes emerged: industry globalisation, cross-industry overlap, customer-product relationship, and attitude towards policy. The industry was described as transnational, generally decentralized, non-cartelized, with *ad hoc* relationships between suppliers, distributors and retailers. Ties with the cigarette industry were apparent. The waterpipe industry appeared to be in an early growth phase, encroaching on new markets, and comprising of mainly small family-run businesses. Customer loyalty appears stronger towards the waterpipe apparatus than tobacco. There was a notable absence of trade unionism and evidence of deliberate breaches of tobacco control laws.

**Conclusion:** The waterpipe industry appears fragmented but is slowly growing into a mature, globalized, and customer-focused industry with ties to the cigarette industry. Now is an ideal window of opportunity to strengthen public health policy towards the waterpipe industry, which should include a specific legislative waterpipe framework. **** ****

## Introduction

Using the waterpipe (also commonly known as narghile, hookah and shisha) for tobacco smoking has been commonplace in Asia and North Africa for centuries. Its popularity has grown in Europe and North America in the last two decades, against a backdrop of a plateauing or decreasing cigarette prevalence ^1– 3^. A systematic review conducted in 2016 showed that the proportion of adolescents who had tried waterpipe tobacco in the last 30 days averaged at 10.6% for Europe, 10.3% for the Eastern Mediterranean, and 6.8% for the Americas ^4^. Past-30 day waterpipe tobacco use amongst youth in Jordan increased from 14.0% to 22.6% between 2008 and 2011 ^5^, with similar patterns seen in Lebanon ^6^, Canada ^7, 8^, and the US ^9^.

A recent review found that waterpipe is perceived as less harmful and less addictive compared to cigarettes ^10^. However, toxicological studies have consistently shown that waterpipe tobacco use exposes smokers to significant quantities of tar, nicotine, carbon monoxide and carcinogens ^11^. One meta-analysis of 17 studies measuring toxicant exposure from waterpipe tobacco showed that a session contains about 4.1mg nicotine, 619.0mg tar, and 192mg carbon monoxide ^12^. Unsurprisingly, epidemiological studies have shown associations between waterpipe tobacco use and several cancers, respiratory diseases, cardiovascular diseases and low-birth weight ^13^. These harms are compounded by the fact that the manufacture, marketing and consumption of waterpipe tobacco is not adequately regulated, particularly when compared to cigarette smoking ^14– 16^. Accordingly, there have been calls for more in-depth research to understand the most effective tobacco policy responses to counter this ^17^.

Little research has been done to uncover the features of the waterpipe tobacco industry. This makes designing effective interventions and policies to counter this expanding trend challenging. A growing understanding of the cigarette industry has been important in advancing tobacco control globally, and the same is needed for the waterpipe tobacco industry.

Our group previously attended a waterpipe trade exhibition in 2014 and showed that marketing material most commonly described waterpipe as a healthier alternative to cigarettes, with emphasis on its flavours, safety, and quality ^18^. Furthermore, we found that transnational tobacco companies were partnered or affiliated with a number of waterpipe tobacco exhibitors ^19^. At the 2014 trade exhibition visit we also demonstrated an overlap between the electronic cigarette (e-cigarette) and waterpipe tobacco industry. For example, the majority of exhibitors displayed e-cigarettes of various sizes, rebranded as ‘electronic waterpipes’ (e-waterpipes) ^18^. Based on an analysis of products found on marketing material, we concluded that electronic waterpipe products were offshoots of the e-cigarette industry competing against the waterpipe tobacco industry ^19^. Whether e-waterpipes are the next evolutionary step in the waterpipe tobacco story remains uncertain.

The objective of this study was to describe the features of the waterpipe tobacco industry. This included an understanding of the distribution of waterpipe tobacco manufacturers, distributers and retailers, identifying unique selling points to products, exploring the concept of brand loyalty and understanding the challenges faced by the waterpipe tobacco industry.

## Methods

### Design and setting

We conducted semi-structured interviews ^20^ with representatives of waterpipe tobacco companies participating in the third International Hookah Fair. The fair took place on 2 ^nd^ and 3 ^rd^ March 2015 in Frankfurt, Germany. The fair organizers described it as “the only trade fair primarily specializing in waterpipes, electronic shishas, hookah tobacco, charcoal and its requisites” ^21^.

### Eligibility and sampling

We used our previously developed waterpipe product categorisation scheme ^18^ to decide on eligibility. We included representatives of companies that sell waterpipe consumption products (tobacco or tobacco substitutes) or waterpipe accessories (e.g., apparatuses, charcoal). We excluded exhibitors displaying only e-waterpipe products, as we aimed to focus exclusively on the waterpipe tobacco industry.

We created a sampling frame by visiting each exhibition stand and judging whether it met inclusion criteria. We marked eligible exhibition stands on an exhibition map and then measured their surface area as shown on the exhibition map. We then aimed to randomly sample a minimum of 30% of eligible exhibitors using probability proportional-to-size sampling, to ensure the representation of exhibitions with varying placement, footfall, and product type.

### Data collection

Three authors (AD/MJ/TL) collected data by approaching potential participants at their exhibition stands, introducing themselves, and stating our objective as conducting a research project on the features of the waterpipe tobacco industry. Researchers took turns to lead each interview but all participated by asking questions and making comments in a conversational manner. Privacy with participants was not possible given the public setting. We adapted the interview guide from a similar study conducted among smokeless tobacco industry players in India ^22^. Supplementary File 1 shows the questions included in the interview guide.

We chose not to audio-record the interviews for practical reasons (e.g. the constant loud music would result in poor quality recordings), and did not have ethical approval to do so. Instead, the data collectors audio recorded their recollection of the discussions outside the main exhibition hall within a few minutes of completion of each interview. All three researchers contributed to this discussion, ensuring the recollection was well-triangulated. 

### Data analysis

We transcribed all audio-recordings from the semi-structured interviews. We used a qualitative approach to identify emerging themes using an inductive approach, drawing on grounded theory ^23^. We applied the following three coding procedures ^24^, but not necessarily in the following order: open coding (whereby we looked for themes and subthemes and categorized responses based upon differences and similarities between responses); axial coding (checking for gaps and overlaps between the subthemes to ensure that each one was fully elaborated); and selective coding (whereby all subthemes were compared and unified around core themes).

## Results

Thirty-three exhibitors met the inclusion criteria. Despite time constraints, we managed to randomly sample 20 exhibitors (61% of all eligible exhibitors). The duration of the interviews varied widely depending on the level of engagement by the exhibitor. Most interviews lasted less than ten minutes, and the longest lasted over an hour.

The following key industry features emerged from the thematic analysis, which we detail below:

1. Industry globalisation
2. Cross-industry overlap
3. Customer-product relationship
4. Attitude towards policy

### 1. Industry growth

Industry globalisation was demonstrated by the suggested that individual components of the waterpipe (e.g. apparatus, tobacco, and charcoal) may be sourced from different countries based on regional material and industrial strengths. The industry itself appears to comprise a host of regional networks and relies on transnational links. Tobacco producers appear to be based largely in low and middle-income countries (e.g., Egypt, India), whilst the apparatus is often made in the Far East (e.g., China) because of lower production costs. A minority of the higher-end waterpipes were made in Eastern Europe because of their expertise with Bohemian crystal and glassware. The most popular form of charcoals in the West are either bamboo-based (usually made in Russia) or coconut shell-based (manufactured in South East Asia). Finally, the distributers and retailers of all products were predominantly based in the Middle East (e.g., in Lebanon, Turkey).

Globalisation was also evident in terms of waterpipe companies reaching out to new markets, both in terms of new regions and new demographic groups. Several waterpipe tobacco distributors mentioned that the European market for waterpipe tobacco has already witnessed growth, but is now stabilizing and potentially reaching saturation. Some interviewees stated that the waterpipe tobacco industry is now encroaching into previously unexplored markets, for instance New Zealand, Russia and Mexico. New pricing strategies would target younger adults with less disposable income, and the manufacturing quality of waterpipes would be lower to accommodate this. That said, we understood that many of the company representatives were family members of the owners, suggesting that small, family-led businesses are still commonplace and the emergence of an oligopoly (i.e. the industry is dominated by a handful of companies) has yet to occur.

The globalisation of the industry was also demonstrated by presence at the convention of international counterfeit products, which was described by two exhibitors. One exhibitor, the owner of a large waterpipe apparatus-producing firm, explained that a main motivation for being at the exhibition was to identify counterfeits of his product. He angrily pointed out an exhibitor displaying waterpipe apparatuses with a similar name, which he alleged was an attempt to counterfeit his well-established brand. He went on further to say that when he approached representatives of this exhibitor, they claimed that the product was named after the owner’s daughter, and not after his brand.

Support of industry growth was seen in the development of new and innovative products. We saw at least three types of charcoal products (briquettes, quick lighting discs, and bamboo/coconut shell-based) in addition to electronic heating elements replacing the charcoal altogether. We witnessed hundreds of tobacco flavours, including a move away from flavour descriptors (e.g. ‘ecstasy’ flavour, ‘twist’ flavour, and ‘green’ flavour) and a growing number of exhibitors displaying tobacco substitutes such as flavoured steam stones and herbal, non-tobacco varieties. We noticed that most of the innovation was by the apparatus manufacturers. In one example, a manufacturer was selling a waterpipe apparatus that had an aquarium with fish incorporated into its base, giving the illusion that the smoke was passing through the aquarium. Several exhibitors were selling ‘diffusers’ – small devices placed on the descending stem of the apparatus which creates smaller bubbles as the smoke enters the water.

### 2. Cross-industry overlap

Interviews suggested a number of cross-industry overlaps, linked directly to the different product types of the waterpipe industry. For example, one of the largest and most well-positioned stands at the exhibition (immediately in front of the main doors), displayed the logos of Al-Nakhla (a leading tobacco manufacturer) and Japan Tobacco International (JTI) on their banner (Al-Nakhla was purchased by JTI in 2012) ^19^. Another company representative revealed that it is now commonplace for his waterpipe company to exhibit at general cigarette and tobacco trade exhibitions; the Dortmund Intertabac exhibition in Germany was directly mentioned, and other exhibitors mentioned exhibitions in France, England, and Poland.

We found no evidence that ties to the cigarette industry were present for other waterpipe manufacturers, such as charcoal and apparatus manufacturers. Instead, these manufacturers were connected with non-tobacco industries; rather than waterpipe companies reaching out to other industries, it was generally felt that it was non-tobacco industries reaching out to the waterpipe industry. For example, one company, a successful barbeque charcoal manufacturer for nearly 100 years, have now become a main player in the waterpipe industry. The owner of a Germany-based waterpipe apparatus manufacturer described how his family have been involved in glass making for generations – in the last eight months he moved to making waterpipe apparatuses after his son started using it.

### 3. Customer-product relationship

Several Exhibitors displaying waterpipe apparatuses explained that the engineering and design of the apparatus were their unique selling points, particularly for more expensive, high-end apparatuses which may appeal to those who see it as a source of pride. One apparatus manufacturer described how his high-end, crystal-based apparatuses were bought by several high profile celebrities. Another were selling their bohemian glass apparatuses for between 149 and 249 Euros each, which was about ten times more expensive than their standard range.

Ensuring high quality for customers was a consistent theme across exhibitors displaying waterpipe products of all types. At least two exhibitors displaying waterpipe apparatuses boasted about how their parts were made of rust-free stainless steel, brass or even more expensive and long-lasting materials. One representative claimed that, in the last ten years, only four of their pipes had rusted, and only because they were scratched or damaged. In another example, exhibitors displaying coconut-based charcoals proudly explained how their products remain hotter for longer compared with traditional briquettes, reducing the ‘inconvenience’ of continuously needing to get up and change the charcoal when it cools. 

Customer loyalty also emerged in several interviews. A more in-depth interview with one exhibitor revealed that customers would routinely ‘shop around’ trialling many different products before deciding which combination they like. A few of the more established companies at the exhibition described brand loyalty resulting from the reputation. The more newly established companies described loyalty towards their particular flavours or charcoal types, rather than loyalty to their company brand per se. In general, we were given the impression that loyalty was stronger towards the apparatus rather than to the tobacco or charcoal, and when looking only at tobacco product loyalty, this was stronger towards the flavour rather than to the company producing the flavour. In one example, warmer apple tobacco flavours are popular in winter, while in the summer months cooler tobacco flavours (e.g., mint) are the bestsellers, according to one exhibitor.

### 4. Attitude towards policy

We asked directly about trade associations and lobby groups, and found these to be absent or severely lacking in the waterpipe tobacco industry. For example, the owner of a major apparatus-producing company described how his company was one of few he knows of that had its own lawyers on board ready for trade disputes and other legal battles, and that other companies preferred short-term financial gains rather than long term legal protection. He explained that this may be because the industry was originally based in the Arab world, where there is a shorter history of trade unionism, political lobbying and a more laissez-faire attitude towards respecting the law.

We found two deliberate violations of tobacco policy. In the first example, a representative of a wholesale retailer described how their companies’ products are priced so that they are middle of the range and affordable. When probed for their specific target audience, this was reported as youth as young as ten years old upwards to those in their mid-thirties. In the second example, we found that some waterpipe tobacco manufacturers exploit the self-assembled nature of waterpipe tobacco smoking to deliberately avoid tobacco ingredient laws. One example that came up separately in four interviews was regarding a German law from the 1970s that prohibited more than 5% glycerin in tobacco products. We saw several instances of companies selling glycerin in separate bottles that end-users could mix into their tobacco to improve the flavor. One tobacco manufacturer at the exhibition explained that 20–30% glycerine was needed to keep the quality of the flavour. Another participant admitted to using more than 5% glycerine in the manufacture of his waterpipe tobacco in order to keep the flavour from being too dry, and said he felt pretty lucky that bypassing the authorities was not creating a problem for him. This indicates that enforcement of these laws may also be lacking.

Dataset 1. Transcripts that formed the basis for this study http://dx.doi.org/10.5256/f1000research.13796.d192937
Copyright: © 2018 Singh N et al. 2018 Data associated with the article are available under the terms of the Creative Commons Zero "No rights reserved" data waiver (CC0 1.0 Public domain dedication).

## Discussion

### Main findings

This study reports the features of the waterpipe industry under four key themes: industry globalisation, cross-industry overlap, customer-product relationship and attitude towards policy. Our understanding is that the waterpipe industry is in an early growth phase, demonstrated by increasing globalisation, reaching out to new markets, the growing presence of counterfeits, and the development of new and innovative products. However, it is still relatively immature, comprising many small companies, often family businesses, who may be specialized in non-tobacco sectors such as glassware and less interested in long term legal protection. A complex web of interactions occurs with neither centralized planning nor cartelized regulation, relying instead on *ad hoc* personal and professional relationships between partner companies. Further, the diversification of products at this early stage may be considered a threat to product loyalty, which in itself is already quite weak. Perhaps the most pertinent finding is that the “waterpipe industry” is multidimensional and difficult to define. The fact that the waterpipe industry is not a single entity, but rather a conglomeration of actors from both tobacco and non-tobacco industries, will make the development of effective public health tobacco policies challenging.

Our interpretation of cross-industry overlap, with particular reference to JTI’s purchase of Al-Nakhla, is that waterpipe tobacco and cigarette industries are interaction with one another. These two smoking behaviours are not seen to be mutually undermining, but mutually reinforcing. This is a clear pattern historically seen with transnational tobacco companies producing smokeless tobacco products in addition to cigarette. Further, our interviews suggested that the waterpipe industry derives much of its legitimacy and endurance from its links with non-tobacco industries. The relaxed view, and sometimes deliberate breaches, towards policy is not unexpected for a tobacco industry; however the fragmentation of the industry across many small companies may make enforcement of policy challenging and resource-intense.

### Previous literature

Largely thanks to the presence of internal industry documents, we know much about the features of the cigarette industry. The cigarette industry has used its economic power, lobbying and marketing machinery, and manipulation of the media to discredit scientific research and influence governments. It has also injected large contributions into social programs worldwide to create a positive public image under the guise of corporate social responsibility. The waterpipe industry appears to mirror and replicates some, but not all, of these tactics. Our findings, in concordance with the literature, show that the most two salient tactics used by the waterpipe industry are deceptive marketing messages, mainly targeted towards youth ^14, 18, 25^ and blatant disregard for nearly all tobacco policies ^15, 26– 29^. However, we are yet to see evidence of lobbying and involvement in social programs, perhaps due to the lack of economic power within the industry. One of the authors (MJ) noted a weak attendance from the waterpipe industry at the 2016 public meeting hosted for the US Food and Drug Administration, where only one company representative from the US spoke briefly against the move towards stronger waterpipe tobacco policy. Furthermore, the waterpipe industry differs from the cigarette industry in several key respects: the large number of small family businesses, the use of reusable apparatuses with user identification and product loyalty centred at least as much on this apparatus as on the tobacco, and the lack of a legal self-protection; how these features impact the waterpipe industry’s ability to emulate the known tactics of the cigarette industry, remains to be seen.

### Strengths and limitations

A key strength of this research is that it is the first, to the best of our knowledge, to use qualitative methodology to explore the features of the global waterpipe industry. In the absence of internal industry documents, deciding to sample company representatives at the world’s largest waterpipe tobacco trade exhibition is an informative first step in developing a greater understanding of this industry. Our findings, taken together with what we know about waterpipe tobacco and the cigarette industry, offer important insights into the development of the industry and potential foci for further research.

This study has several limitations. Those attending the trade exhibition in Germany may not represent the global waterpipe tobacco industry; rather we suspect our sample was more over-representative of German waterpipe companies given its location. While waterpipe tobacco use is highest in Middle Eastern countries, that this fair was targeted to Europeans is an important finding. It is possible that this fair was more representative of waterpipe companies that place importance on such marketing events rather than those who do not; this may also make our results non-generalizable. Not knowing which companies will be attending the event *a priori* limits our capacity to get background information and assess the importance of these companies before meeting their representatives. Our interviews were not recorded on tape, and we instead relied on researchers’ recall and interpretations of discussions to retain the key points made. While this could introduce recall bias, we made all efforts to record the information within minutes of the interview to maximize recall, and every interview was attended by three researchers, minimizing the possibility of bias. Finally, introducing ourselves as researchers may have influenced participants’ responses. However, the nature of the event was such that networking and conversations were encouraged, and we did not expect more “truthful” answers had we not introduced ourselves as researchers, given we were unacquainted with our participants anyway.

### Implications

Understanding the *modus operandi* of the waterpipe industry can help design effective interventions and policies to counteract the increasingly widespread use of these products and its potential implications for public health. Given the vast number of small businesses in the sector, now is an ideal window of opportunity to strengthen public health policy towards the waterpipe tobacco industry. However, given the waterpipe industry derives much of its legitimacy and endurance from its links with non-tobacco industries, interventions aimed solely at tobacco are at risk of failing. Cigarette regulations will likely not be effective at controlling waterpipe tobacco use, since they are aimed at targeting large, established companies that mostly use traditional means of advertising to promote the purchase of their products from supermarkets and other regulated vendors. Further, considering waterpipe-specific charcoal manufacturers commonly market their products as ‘healthy’ or ‘healthier’ than cigarettes ^18^ despite their highly toxic emissions ^30, 31^, we re-iterate previous calls to treat charcoal products designed for waterpipe tobacco as a proxy tobacco product. We therefore echo calls for a specific legislative waterpipe framework to be developed that accounts for these unique aspects of the industry ^32^, and a call for licensing of commercial waterpipe-serving premises in a similar fashion to the alcohol industry ^15^.

We have identified several important research implications. A myriad of tobacco and tobacco-free products were marketed and sold side-by-side at this fair ^19^, indicating the need to assess whether non-tobacco products are a gateway to future waterpipe or cigarette tobacco. We tried to look indirectly into the workings of the waterpipe tobacco industry, but more work needs to be done to confirm our findings. We call for additional qualitative research to gain ethnographic information on waterpipe tobacco users, sellers and manufacturers similar to the insights into the workings of the cigarette industry ^33^. Pressing questions include the need to identify the main players in the waterpipe industry, their market shares, and their influence on the supply and demand chains, if any. Given the pivotal role of Global South countries in the production and distribution of waterpipe tobacco products, the specific impact of waterpipe tobacco consumption in the West on developing countries is also a question that warrants asking ^34, 35^. A close collaboration between social scientists and public health researchers is needed to fully understand the political economy of the waterpipe tobacco industry.

## Conclusion

To conclude, this qualitative study has provided insights into the waterpipe tobacco industry structure and features. Policy-makers could benefit from these findings when designing waterpipe tobacco-specific interventions, to curb the rise of waterpipe tobacco-related disease.

## Ethical statement

The Imperial College Research Ethics Committee approved the study (Reference: ICREC_14_3_6). Written informed consent was not obtained for participation in the study as it was designed as covert participant observation.

## Data availability

The data referenced by this article are under copyright with the following copyright statement: Copyright: © 2018 Singh N et al.

Data associated with the article are available under the terms of the Creative Commons Zero "No rights reserved" data waiver (CC0 1.0 Public domain dedication). 

Dataset 1: Transcripts that formed the basis for this study. DOI, 10.5256/f1000research.13796.d192937 ^36^

## Supplementary material

Supplementary File 1: Questions that guided the semi-structured interviews.
