# Supplementary material for: Features of the waterpipe tobacco industry: A qualitative study of the third International Hookah Fair
Source: F1000Res. 2018 Oct 23;7:247. Originally published 2018 Feb 28. [Version 2] doi: 10.12688/f1000research.13796.2 (PMC6347030; doi:10.12688/f1000research.13796.2)
Supplement: Dataset 1. Transcripts that formed the basis for this study — http://dx.doi.org/10.5256/f1000research.13796.d192937 [file f1000research-7-18271-s0000.tgz › ecc7f7b5-80fb-4867-b6af-b2c86596220c_Dataset_1._Transcripts.docx]

**Transcription of Audio Files**

**Recording No:** 69

RESEARCHER 2: So Company A, they are a wholesale retailer business.

RESEARCHER 1: They are a manufacturer of waterpipes?

RESEARCHER 2: They are a manufacturer as well, mainly of Shishas, which are set up in Europe but then they send them to Egypt. They are mainly located in Egypt, the guy we talked to was a purchaser. They don't deal with end users.

RESEARCHER 1: They sell to retailers and wholesalers, so they are a manufacturer and distributer.

RESEARCHER 2: They weren't really affected by their competition, because they have a target audience, which are people who are looking for a price range that's kind of in the middle, affordable. They range between 10 year olds and 38 year olds

RESEARCHER 1: So you said the market has stabilised over the past couple of years, it hasn't really increased or decreased, especially in Germany...

RESEARCHER 2: Yeah, they are spreading out to New Zealand, Russia, Mexico, but their general distribution is in Germany. They have affiliations with Company B, and Company C, right?

RESEARCHER 3: They also do their own accessories

RESEARCHER 1: They have a diverse range of products which he agreed contributes to the stability of the organisation, that's one of their strategies, to be broad. But their focus is waterpipe apparatus manufacturing. And the features of that were: stainless steel; doesn't rust; high quality

RESEARCHER 2: In ten years only 4 had rusted and that's because they were scratched.

RESEARCHER 3: So anti-rust was a unique feature.

RESEARCHER 2: They advertise their products through exhibitions and fairs, like in the US...

RESEARCHER 1: You mentioned InterTabac, which is in Dortmund similar to this one as well. That's the big, general tobacco exhibition. So waterpipe companies are in the general cigarette exhibitions as well, which is interesting...

RESEARCHER 2: So their main benefit is that they are affordable, they are high quality, they don't really have main challenges because they figure if other people do things better, then they might as well hand them that. They are the first to bring tobacco shisha.

RESEARCHER 1: They've been around, they are established, and that produces loyalty,

RESEARCHER 2: What was the loophole again?

RESEARCHER 1: So, there's a law from the 1970s saying that you are not allowed more than 5% glycerine in tobacco, so they get around that by putting the maximum amount of glycerine in it, and then selling additional glycerine in separate bottles for the user to put in at home. Which is interesting.

RESEARCHER 2: Once they are sold it's not their business if people are taking in unhealthy products. They've been in the business since 1979, so the owner of Company A started out in 1979 and the purchaser has been in the business for ten years.

RESEARCHER 1: All their products had health warning labels, some were smaller than others but they all had them on the side or front. He mentioned something about same level of taxation as piped cigar/tobacco, which is what they pay. They have issues around not covering the tax label on the packaging. I wouldn't expect them to get into much legislative difficulties as they are manufacturers as opposed to sellers. If they were retailers...

RESEARCHER 2: They would experience more challenges.

70

RESEARCHER 2: They are charcoal, they have 5 different types, they have Belgian charcoal (8 boxes on display), there was a type called FortyFour, there were 4 boxes on display, Koala charcoal, there were 16 boxes on display, Company A Bamboo 8 boxes, and Company D 20 boxes (10 that were 1kg, 10 that were 3kg).

RESEARCHER 1: They had a whole cabinet full of tobacco, on the first layer they had a Karhoo 50g tobacco which had 30 of them on there, the next layer had the 200g version of which there were 12), the layer under that had Miso and the Company C ones, there were 17 of them, and the bottom had a variety of cocktail ones, there were 14 of them. I also saw tobacco substitutes in the form of Hookah squeeze and Ice Rocks, and the additional glycerine, they were selling plenty of them, in addition to the water flavouring powders as well.

RESEARCHER 3: As for the apparatuses, there was 107 on display, 79 of them were only in the middle of the display, the others were dispersed so that people can actually try them, they were all the same type except the number of hose that go in, some would have only one, others have up to 4.

RESEARCHER 1: They also had random accessories like charcoal heaters...

RESEARCHER 3: The pumps....

71

RESEARCHER 2: So, Company E, and Company F – they are the same – are manufacturers, wholesalers and retailers. They make their products in the Emirates, then bring them on to Germany and Europe in general. Everything else is done in Germany. He is the purchaser, the manager for sales. So who buys their products? They have a very wide range of products, and their price range is very wide so they are able to capture all people. They have more than 100 flavours, they have 3% glycerine in their tobacco.

RESEARCHER 3: First he said 'The legal amount', then he said 'I think it's 3%'

RESEARCHER 2: Yeah, he wasn't very sure. They also sell the liquid so that they can be added at home, the same as with Company A. Company E has only been there for one year, and they feel that they have been doing extremely well compared to other companies that have been on the market longer, that's why they don't feel very threatened, because they accelerated at a very high speed.

RESEARCHER 3: The customers are not limiting themselves to one type. He said that customers are always trying to change the brand especially in Germany so that's why they are doing well.

RESEARCHER 2: They try to advertise their products in exhibitions here, but there's also exhibitions that are going on in London and France.

RESEARCHER 3: And in Poland this year.

RESEARCHER 2: What is their most popular product? 'Everything', but especially the 'Ajamay' one. Tobacco. How do make sure the consumer buys from you and nobody else? They really don't feel they need to be competitive because people are very curious, so they always want to try something new. They are affordable, and they think they are of high quality as well. Unique selling points? They are mainly known for their Ajamay. Do you have a range of diverse product types? They do they mainly go to trade fairs and exhibitions. Their suppliers are based in Germany and dealing in Europe, not sure about outside Europe. What are the main benefits to selling your product? They have just started up so they don't feel like they have faced many challenges yet, they are doing very good for a start up.

RESEARCHER 1: When you say they are a wholesaler, retailer and manufacturer, do they have a shop in the Emirates?

RESEARCHER 2: No, they don't sell anything in the Emirates. They are manufactured in Emirates and sold in Emirates. They sell to the end consumer. But who do they deal with, he didn't say, he didn't want to get into those details. He was a little resistant, rushed, a little disoriented, someone else came in so he wasn't very focussed on us.

72

RESEARCHER 2: Ok so Company E had only 2 boxes of charcoal, 1kg each, and the type was CoCo my Gold.

RESEARCHER 3: And 40 apparatuses.

RESEARCHER 1: And the tobacco … they had 74 of the 200g big tubs of tobacco, they had 33 boxes of the 20 in a pack of the 30g, they had 17 individual 30g ones as well. And they were all Al Ajamay with a range of flavours.

73

RESEARCHER 2: Do we want to talk about the Company G

RESEARCHER 1: Yes, they were on the same stand together.

RESEARCHER 2: There was Company H and Company G. Company G are working with Shisha, they produce the glass. And everything in the apparatus. They are manufacturers in Germany. They were the owners of the company. Who buys their products? Company H mainly...

RESEARCHER 1: They also mentioned some wholesalers that they sell to, I couldn't catch their names but there were at least 4 or 5 that he was reeling off, in Germany, and their rate of sale has been consistent, they have been around for 8 months.

RESEARCHER 2: And what's helping them is that they have a glass company.

RESEARCHER 1: Which is interesting because he is still making other glass products and now water pipe is, for him, just a diverse product range to make him stable.

RESEARCHER 2: He smokes cigarettes and his son is a shisha smoker, and his son showed him, and helped him come up with the business idea. What's good about them is that if any of the pieces are ruined they can re supply (that bit).

RESEARCHER 3: Whatever is broken they can give you another piece, they can provide spare part for every part of the shisha

RESEARCHER 2: So far they are doing really well but they haven't been in the market long enough to see a fluctuation.

RESEARCHER 1: That's not the first time that we have interviewed someone who had been around for a short time.

RESEARCHER 2: Company A are the only ones that have been on the market for very long. And that's probably why they had so much emphasis on sheeshas. But otherwise people are working on diversity and … Ok we'll get to that in a bit.

RESEARCHER 3: There's Company H, and Company I, there's both.

RESEARCHER 2: They are manufacturers, but they are also retailers and wholesalers. Right?

RESEARCHER 1: He distributes to wholesalers and retailers.

RESEARCHER 2: They are mainly located in Germany but they also deal all across Europe, but also a little bit in Israel and the USA. They are relatively cheap, so they don't do discounts, they work on the fact that they can provide service, so it's all online: you order, you get it the same day, a business model like Amazon's. They have been around for 12 years, doing pretty well, they stand out. They were one of the first in Germany. Spare parts … he said that being in the exhibition is expensive but the main reason they were there was they wanted to do their PR, maybe some partnerships. And hopefully break even.

RESEARCHER 1: He talked a lot about the glycerine content again, about legislation issues,

RESEARCHER 2: Except this one was different, he was ok being in the grey zone. Some of the tobacco products have 20-30% of glycerine inside, but it depends on the season, sometimes its dry sometimes it’s wet. When it’s dry they do their mixing with the liquid gel. And it’s a bit of a challenge getting it through but generally people don't look at the details so they don't look at the percentage

RESEARCHER 1: The authorities don't look at the composition. So that's, not a loophole, but a failure of enforcement really.

RESEARCHER 2: He said he felt pretty lucky that it was bypassing the authorities and not creating a problem for him, but sometimes end users are not very happy, or the retailers/wholesalers are complaining when it’s too dry. So that's why they're coming up with alternatives.

RESEARCHER 3: With the liquids and the gels

RESEARCHER 2: So two or three days and they have a perfect mix.

RESEARCHER 1: So as a manufacturer, it’s manufacturing the flavour of Mo’assel tobacco, and that has its challenges in terms of getting the taste right for the end user. His challenge is the taste of his products

RESEARCHER 3: That's when it comes to selling it in Germany, because of the law, its fine selling outside Germany.

RESEARCHER 2: What are you talking about, we're talking about the flavours. He tries to stay stable with the quality of the flavours and the satisfaction of the people buying his products.

RESEARCHER 1: He had 174 tubs of the 200g flavoured tobacco, and 40 of the 25g unwashed tobacco.

RESEARCHER 2: He had 160 boxes of charcoal. Company D. The 1 kg boxes. Those are the ones that weren't sold, and we're almost halfway through the day.

RESEARCHER 3: 39 apparatuses. A couple of them were very different, they had an aquarium inside or...

RESEARCHER 1: Do you actually use that to smoke water pipe

RESEARCHER 3: I think so because the one in the middle was shaped really weirdly with a lot of coloured liquids in it.

RESEARCHER 2: I think it also looks good so people who are smoking at home can use it as an accessory but also use it.

RESEARCHER 1: So far all of the charcoal we have seen has been Bamboo right? Company D…That's important. Some were coconut too.

74

RESEARCHER 2: He wasn't very clear what else he gets involved with. He gave us examples of everything else that he does but he wasn't extremely clear about it... who knows if he is involved in e-cigarettes, we didn't get a clear answer on that.

RESEARCHER 1: It didn't sound like he was... He is a glass maker. That's what he does. He creates high-quality waterpipe apparatuses. He says he owns the original patent of “blablabla” since 1820. They have 26 patents in the US. His name was HN.

RESEARCHER 2: That's a Syrian name, but we don't know if that's his family name. It's called Company J, otherwise known as Company K in Lebanon. And it was located in Place A in Lebanon, near the syndicate of doctors.

RESEARCHER 3: There was also a hospital...

RESEARCHER 2: It started out in 1863 as a family business. It was the first company to manufacture arghiles in China. They have 26 patents in the US.

RESEARCHER 3: It started in the 1800s...

RESEARCHER 2: The German market started with a glass pipe with a Hookah. So they are merging two different concepts with each other. He talked about Jordan and the way different places call it differently. So we (Lebanon call it Arghile, Jordan call it hubble bubble)

RESEARCHER 1: We call it Shisha (UK), in America the thing is called Shisha

RESEARCHER 2: And in America Shisha is thought of as the molasses only.

RESEARCHER 2: He also talked about Company L as being one of the big manufacturers. Especially in the UK.

RESEARCHER 3: Company L produces Mo’assel?

RESEARCHER 1: No they produce the pipe as well.

RESEARCHER 2: He says their main unique feature was the brass steel.

RESEARCHER 1: He bought high end, premium crystal apparatuses for high end customers: Celebrity A, Celebrity B’s daughter, Celebrity C.

RESEARCHER 2: They had offers to do business with Porsche but they turned them down.

RESEARCHER 1: I found out that ties with the cigarette industry only really happens among the tobacco producers, not really among the pipe producers. It seems like it is very clear that a company that produces the apparatus is probably also producing the chandeliers and the other vases, and the other glass products, but they are not really involved in the tobacco part. It's the tobacco guys who are producing the Mo’asel and the glycerine. What we haven't seen yet is, and we haven't spoken to these guys, the charcoal companies probably have nothing to do with the cigarette companies either, but they produce barbeque charcoal and waterpipe charcoal. I'm just trying to get a handle on how the industry is structured. It seems to be in three layers: the apparatus producers, influenced by the drug industry producing bongs as well; the tobacco producers, who are influenced by the cigarette industry; and the charcoal producers who also produce BBQ charcoal. So it’s a three-tiered industry. Company L, Company K is said to be owning the apparatus part, Company B is one of the main tobacco producers, and Company D are dominating everything with all these coconut things. I think he's annoyed that there's a lot of competition now. China undermining his work...

RESEARCHER 2: He's annoyed that he was here last year but he's not this year.

RESEARCHER 3: Maybe that's why he is here, to see who else is here

RESEARCHER 1: I think he is here just to check out these brands. The level of counterfeit copying, I think is high

RESEARCHER 2: Despite the fact that they have the patents, they can't stop people in China from copying. And everyone has the Company K form. And they all look the same. That's one of the biggest things.

RESEARCHER 1: Legislation: so he talked about US health policy, he's got lawyers that protect him, but the industry doesn't. I think it’s such an important finding that there is no trade association that has any voice, because they are Middle Eastern. And because Middle Easterns are a bit less educated, are not western educated, they are not on it.

RESEARCHER 2: He talked about the arms and tobacco forces. He talked about getting grants from Phillip Morris.

RESEARCHER 3: He also talked about how, in order to get his products into the US, he has to sign a contract to make sure no one else gets his products in the US.

RESEARCHER 2: He was talking about being covered, so that he can always have a market for his products. His products are already accepted and approved.

RESEARCHER 1: His Company K is strong brand loyalty he has got, that's what he relies on. That's his selling point.

75

RESEARCHER 2: Company M, they are a factory based in Indonesia. They have a distribution company in Lebanon. They are manufacturers and they export to manufacturers as well. They rarely deal with end-clients or retailers or wholesalers. Their charcoal is from the coconut shells and its 100% coconut shell. They are not the first to use coconut shells to make charcoal, but Company M were the first to branch out internationally in the way they have. Their benefit is that there's no cutting trees so it’s ecological. While others were manufacturing activated carbon. Coconut based charcoal lasts much longer than tree based charcoal, cost are less, it lasts longer, and that means you don't have to keep getting up. The smell is less annoying, less prominent, the smoke production is less, it's sustainable. They are trying to make a deal with Company D for distribution. Right now they are very strong in the MENA region and in the US, and they have partners in the US for flavoured tobacco. They are trying to branch out right now into Russia and Asia and Europe. Pricing and legislation we need to ask about tomorrow. The wife believes it was started in 2006. Other than exhibitions and fairs, they advertise their products through social media. They also cover tobacco. They have 18 charcoals on display.

RESEARCHER 1: And 24/25 tobacco in total, they had 3 30g pack, can't remember the breakdown.

RESEARCHER 2: Company N. The boss is Turkish they are manufacturers, they manufacture their own tobacco. They use strong flavours. Their tobacco is mainly distributed in Germany and Austria. Their sheesha on the other hand is everywhere (the whole apparatus), the Company O. We spoke to the retail sales manager. They manufacture tobacco, pipes, they are also distributors. Their Company AA is a bestseller. Their arghile is well known as well. Their unique feature is the brass. Soft air flow. And bohemian glass. They range between the premium price of 149-249 Euros, their economy line is made of aluminium and is lighter and cheaper, and it runs between 20 Euros for the smaller ones to 49 for the larger ones. They have been in the business for 5 years. Their challenge is legislation related. Related to the 5% glycerine limit in tobacco. That's where liquid form comes in. Company P. Their Cactus Lemon is their big one, we spoke to the CEO. They produce in Iran, they have a company in Germany. Since they are manufacturers, they only deal with wholesalers. Paying the retailers less out of favours... They have the Company G partner. Their 50g tobacco is priced at 4.90 Euros and their 250g is around ?18/80 Euros.

RESEARCHER 1: That's a standard price.

RESEARCHER 2: Company Q… sales, the guy we spoke to has been in sales for two years. They sell a diverse range of products, they have the AMY shishas, also made of brass, one their best sellers, they deal with wholesale and customers.

RESEARCHER 1: His words were 'we buy from other people'. I've seen their website, tis just loads of other companies on their website. And then we sell that.

RESEARCHER 2: And their end customers are shisha bars and customers.

RESEARCHER 1: They are wholesalers and retailers.

RESEARCHER 2: Their AMY brass shisha weighs around 20kg. And is about 75 Euros. They have the click-in system, which is new. Tabac honey and the Lime citrus are their best sellers

RESEARCHER 1: And ?hops produce good smoke which is important.

RESEARCHER 2: Yeah there is smoke evolution, and it tastes better.

76

RESEARCHER 3: Company R. They do the tobacco. The Mo’assel. They have 69 flavours, they are Indian. They've been selling all around the world, 16/17 countries. They are trying to sell now in Germany. He wasn't very pleasant or responsive.

RESEARCHER 1: That might have been due to our approach, journalistic and overly direct maybe.

RESEARCHER 3: They do have charcoal, we didn’t talk about that. They have the natural herbs one [herbal tobacco] and the regular tobacco.

Researcher 1: They are a manufacturer from India that sells to importers. I didn’t catch what their best flavoured product was?

RESEARCHER 3: Yeah he didn’t, he was like ‘check our brochure’

RESEARCHER 1: It was definitely a tobacco product, that was the most popular.

RESEARCHER 3: You pointed out one [flavour] that’s called ecstasy.

RESEARCHER 1: He has a lovely range of flavours, which I’m sure will make our paper.... Maybe we could get a list of all the flavours from all the stores we’re going to, and make a list of flavours we came across – 100s probably. He wasn't forthcoming about challenges but again that’s probably down to us. The whole thing of being over the counter, it just ruins the relationship. It becomes just customer versus seller as opposed to with HN where it was more a close, human relationship and there was no counter between us.

RESEARCHER 1: Let's talk about Company S,

RESEARCHER 3: Reminds you of Company K, who do the pipes

RESEARCHER 1: Good point! Yeah, Company S, Company K...I didn’t pick up on that!

RESEARCHER 3: They are manufacturers of the shisha itself. They do all the pipes, they use glass and the metallic parts, they have spare parts for everything. It's very similar to Company G, they said the same thing: that what's different about them is that you can change every part.

RESEARCHER 1: With apparatus manufacturers, the quality of their product is about the kind of engineering of it: how it fits/screws, making share the airflow…it’s airtight. What makes his unique is that it doesn't need a valve for the hose, why that's important I don't know. It's more like a “plug in any hose” pipe thing... you can add an extension to the pipes, you can add in more parts to make the shisha pipe bigger which is a unique feature. They sell to distributers, and his price comes in at 60 Euros each for a distributer. Like Porsche, he has premium ones and slightly less premium ones, but the cheapest is 60, which is high.

RESEARCHER 3: It’s quite close to the price of AMY [waterpipe apparatuses], you know the one we saw yesterday?

RESEARCHER 1: I suspect he has a lot of competition from Chinese making the cheap aluminium pipes probably seen there in front of us. But again that might be a different market. I suspect that people who are going to spend more on expensive pipes do so for a reason. That’s because they may be more cultured, more picky, more experienced workers, more fancy. So it might be that they are just targeting a different market. Maybe we could look into their target audience a bit more. Of course, they’re a manufacturer so they’re selling to a distributer who then gives it to a retailer who then sells it to a customer. So they’re not interested, really [in a target audience]

RESEARCHER 3: You can feel the difference. There are those selling the Chinese parts, just like the Indian ones we’re seeing. You can see the Company S or the one with the Company G, you can see they are at the same level, but they are still not the level of Company A. You know, you can see that difference. And each has its target audience.

RESEARCHER 1: Maybe the younger student with less money…

RESEARCHER 3: …would be able to afford the cheaper ones. Maybe.

RESEARCHER 1: But the audience here is mainly young students. So is his store busy, is that reflective of the target audience? It’s still early days [i.e. 9.30am, fair has just opened], but if you look at how busy each store is and that’s showing which audience is attracted to that, which target audience is attracted to that, if lots of young people are going to his store .

RESEARCHER 3: But who ends up buying? It's all about who actually ends up buying it.

RESEARCHER 1: True, he's a B2B seller, he’s selling to businesses, he's not making sales today is he? Because of the nature of his business, he is a manufacturer, and a lot of these guys here are just customers wanting to buy stuff, Ok let's move on.

77

RESEARCHER 1: Company T. They are trying to make it clear that they are relaxed. They sell to customers and retailers. I put them down as a wholesaler, they buy in bulk and they sell to customers and retailers. They sell only particular brands themselves: Amy pipe, Amy Gold tobacco. Whether Amy is only sold by them and no-one else we don't know yet. Probably. One of their interesting products was the skull based waterpipe apparatus, of which there are only 15 sold at this fair, they are specially made for this fair, priced at about 150 euros, premium price.

RESEARCHER 3: They have all prices.

RESEARCHER 1: They have cheap and premium products. Which isn't a strategy, everyone is doing that. Maybe that’s what HN was talking about. They just sell, sell, sell. They are just after the money. They have lots of flavours.

RESEARCHER 3: I spoke to Company U. On their advertising papers they said retail and wholesale. According to the guy I spoke to they only sell to end customers, but if anyone wants to sell their products they are willing to try. They are Egyptian, they have different brands of the tobacco itself, they have a lot of flavours, and their best selling tobacco depends upon the season—in the winter they sell the two apples, the Tufahteyn and the Anab, the grapes, and in the summer they sell whatever is icy, like ice-mint and that stuff. They have had trouble entering the Arab market. They tried entering the Turkish market and it was not a success, so they sell best in Europe. They have not yet started selling in Germany, they are trying. They produce their own tobacco in Egypt. And their apparatuses are made in China. They have been around for 10-12 years.

RESEARCHER 1: The seasonal variation is important.

RESEARCHER 3: He is the first one talk about it.

RESEARCHER 1: Maybe we can ask the others directly about that, if the ice ones sell more in the summer, to validate them.

RESEARCHER 3: But we might be biasing them that way?

78

RESEARCHER 1: Company V, the Russian guy.

RESEARCHER 3: They have 5 shapes, they manufacture their own in Russia. They were selling very well in Russia, covering 200 restaurants, until laws came up so they had to sell somewhere else. So they are trying to sell their products here. They only sell the Shisha apparatuses. They have something different: the water bowl goes down and the stem is shorter but it ends with a diffuser, as he explained. The diffuser produces smaller bubbles and hence stronger smoke.

RESEARCHER 1: It cools down the smoke, plays a role in filtration. A stronger smoke?

RESEARCHER 3: As in a stronger feeling.

RESEARCHER 1: It is a premium price. The cheapest one is something like 150 euros, going up to 300 Euros, they sell to wholesale mainly, and also to retailers, but today he is selling to the end customer.

RESEARCHER 3: You can order online.

RESEARCHER 1: So that was very interesting. Again, the main challenge he has is the Chinese counterfeiting. That is common among the apparatus manufacturers.

RESEARCHER 3: HN explained that the Chinese have the smallest stand in this Expo, but somehow they are making at least 60% of what is we are seeing. So 60% of people exposing their products here, are actually getting their stuff from China.

RESEARCHER 1: Even the manufacturers who say they are German are probably sending their stuff to China. Probably too expensive to manufacture in Germany. The second guy was global molasses, my friend from the UK.

RESEARCHER 3: He has a list of the molasses that he distributes,

RESEARCHER 1: He distributes for Company W and Company X and Company B and all the major ones, from Dubai. He's a distributer basically. He talks a lot about the UK being renowned for its black market. So they don't export there because it's so difficult.

RESEARCHER 3: He sells worldwide. They have been around for 15 years.

RESEARCHER 1: Not much interesting. Just a distributer.

79

RESEARCHER 1: The last 2 were quite busy. We had Company Y and Company Z, they were both coconut.

RESEARCHER 3: We have a picture of how they are turning the coconut into charcoal.

RESEARCHER 1: All the coconut ones seem to be the same message: lasts longer, less fumes, less odour, less ash.

RESEARCHER 3: Did he use the word healthier?

RESEARCHER 1: No, I don’t think he did. But he said that the composition differs between companies, as does the price. His are slightly cheaper than Company D. Of all the charcoal we’ve seen, it’s been almost exclusively coconut and bamboo.

RESEARCHER 3: There is no wood charcoal here

RESEARCHER 1: No, and I haven't seen the discs (at all/much) either, so this seems to be the craze now. Very important that that's shifted.

RESEARCHER 3: I think the discs were at Company R, the Indian guy?

RESEARCHER 1: We’ll double check and catalogue them. Ok nothing else to add, we’re saturated.

RESEARCHER 1: Arabia, very busy, one guy running the stall, he sells tobacco only as his product, he is a manufacturer of tobacco from the emirates, Dubai.

RESEARCHER 3: He sells in the UK and he is trying to get into Germany but the laws are different in Germany, so he will have to work on that. Regarding the glycerine right? But they don't say how much glycerin is in it. They gave us an empty box. The samples did not have any health warning labels on them, but some of his items did. We have a picture of that.

80

RESEARCHER 1: Perhaps slightly saturated by Company AA. The person was a hostess, they are a manufacturer of tobacco in Germany, they sell to wholesalers, to retailers, to customers, they focus on the taste of the tobacco, they have a new line/generation. The person was a hostess so it was difficult to get information out of her.

81

RESEARCHER 1: Company AB. An American based company, popular outside the UK. A manufacturer of tobacco. His reasons for high quality include: his own recipes and flavours, not copying anything from the Middle East, so original and genuine, and in his words, high quality tobacco leaf. Based in Texas. That’s all I could really get. Lots of flavours.

82:

RESEARCHER 1: Company AC, manufactured in Holland, they manufacture the cannabis flavoured steam stones and the juice that goes with it. You can use the steam stones several times and top it up once the flavour goes with the juice. So it’s a tobacco substitute. They are a manufacturer who sell to customers and retailers, and distributers. They cost 5 euros each.

83:

RESEARCHER 1: So this guy is Company AD, who is a furniture decorator of retail shisha lounges. So selling to retailers only. Also sells pipes. He has 15 pipes on display, manufactures in Germany, sells to retailers, limited English so difficult to engage with. Doesn’t sound too exciting. Probably not relevant to us.

84:

RESEARCHER 1: Company AE. Manufactured in the US. Israeli and Lebanese partners. They distribute to retailers and customers. Their selling point is the disposable head. Six in a pack, 3 Euros per head, 3 in a pack. 18 in total? The whole point is it is hassle free to make the head, he puts 8g of herbal non tobacco in it and he says it is better for retailers, as they can keep an eye on how much is being used, because 8g per head of this herbal stuff. 10 -20 flavours or more.

85:

RESEARCHER 1: Company AF. Waterpipe manufacturer in the US. Make stainless steel pipes, rust-free pipes, that's their selling point. Hand-made glass bowl. Washable hose. Problems regarding Chinese manufacturers copying. Middle priced 50-100 Euros for the end user.

86:

RESEARCHER 1: Company AG. Nothing new, saturated. Another coconut-producing manufacturer of coconut charcoal from Indonesia. He says it lasts about 40 minutes to an hour, maybe. And that other people are exaggerating. But again nothing new. Same thing about no ash, no odour, no nothing.
