# Supplementary material for: Features of the waterpipe tobacco industry: A qualitative study of the third International Hookah Fair
Source: F1000Res. 2018 Oct 23;7:247. Originally published 2018 Feb 28. [Version 2] doi: 10.12688/f1000research.13796.2 (PMC6347030; doi:10.12688/f1000research.13796.2)
Supplement: Supplementary file 2 [file f1000research-7-18271-s0001.tgz › 07bb623c-f1c0-4020-9753-f9438336d7b8.docx]

**Supplementary File 1:** Questions that guided the semi-structured interviews

| Q1: What country are you based in?  Q2: Who buys your products?  Q3: What countries are your suppliers based in?  Q4: What incentives do your suppliers give you to encourage you to buy more from them?  Q5: What is your most popular product?  Q6: What is your unique selling point?  Q7: What challenges do your business face?  Q8: How do you ensure brand loyalty?  Q9: What range of products do you sell?  Q10: What are the main benefits of your product over those of your competitors? |
| --- |
