# Supplementary material for: Features of the waterpipe tobacco industry: A qualitative study of the third International Hookah Fair
Source: F1000Res. 2018 Oct 23;7:247. Originally published 2018 Feb 28. [Version 2] doi: 10.12688/f1000research.13796.2 (PMC6347030; doi:10.12688/f1000research.13796.2)
Supplement: Peer review history

# Peer review: Referee response for version 2

**DOI:** 10.5256/f1000research.18271.r43073

**Published:** 2019-01-23

**Authors:** Mohammad Ebrahimi-Kalan

**Affiliations:** 1 Department of Epidemiology, Robert Stempel College of Public Health, Florida International University (FIU), Miami, FL, USA

Thank you for the opportunity to review the manuscript entitled “Features of the waterpipe tobacco industry: A qualitative study of the third International Hookah Fair ”. This is a well-written and interesting topic that can help policymakers to curb waterpipe epidemic. The manuscript conceptual framework is particularly new since not many research has been conducted on a constructive paradigm (qualitative methodology).  This manuscript has been peer-reviewed by other two collogues who are expert in the waterpipe tobacco field. Hence, I think the manuscript has improved tremendously. I have a few minor comments as follows:

**1. First Paragraph in the results section: "**Most interviews lasted less than ten minutes, and the longest lasted over an hour". This part needs more clarification. I suggest having percentages/ or numbers representing the interview time. For example ?% (less than 10min) and ... 

 2. Usually in the fairs like "International Hookah Fair," some representatives had waterpipe with charcoal on and atmosphere may include the smoke. If the same happened in this study, I would suggest having a sentence in the limitation section to explain this issue that may affect the interview process. 

I have read this submission. I believe that I have an appropriate level of expertise to confirm that it is of an acceptable scientific standard.

# Peer review: Referee response for version 1

**DOI:** 10.5256/f1000research.14998.r33008

**Published:** 2018-05-11

**Authors:** Randah R. Hamadeh

**Affiliations:** 1 Department of Family and Community Medicine, College of Medicine and Medical Sciences, Arabian Gulf University, Manama, Bahrain

This is a very well written  original and interesting article. However, I have some comments and queries:

 Abstract: Results: “20 representatives”, I suggest moving “20” to methods before “representatives”

 Introduction:

1. Para 1: Specify time for this statement“ The proportion of adolescents who have ever smoked waterpipe tobacco was between 4 and 33% in Africa and Asia, between 6 and 11% in the USA, and up to 38% in some UK communities” or, if it is the latest available data, replace “was” by “is”.
2. Para 2: “about 4.1mg nicotine, 619.0mg of tar, and 192mg of carbon monoxide” remove “of”
3. Para 3:“This makes designing effective interventions and policies to counter this growing trend challenging. A growing”,  I suggest replacing the first “growing” by “expanding”
4. Para 3: I suggest moving the 2014 exhibition information to the next para as it is discussed as well.

Methods:

1. Written consent was not taken from the participants and this should be stated.
2. Since the audio recording was done following the interview, the authors should address recall bias and whether consensus between the three data collectors was necessary.
3. Three researchers were present at each interview but did they all participate?
4. How did you ensure privacy if others were present at the exhibition stand ?

Results:

1. Page 4, last para” “Rather, these manufacturers were connected with non-tobacco industries; rather than waterpipe companies reaching out to other industries..” rephrase by removing one of the two “rather”
2. Page 5, Attitude towards policy, line 4: “camping”? Correct to “ company”

Discussion

1. Page 5, last para: "Our interpretation of cross-industry overlap is that waterpipe tobacco and cigarette industries are not in competition, but rather in collaboration.",  The conclusion is not supported with the results.
2. Page 6, para 1: “Furthermore, the waterpipe industry differs from tobacco in several key respects”, shouldn’t tobacco be cigarette industry?

Transcripts

 Authors need to explain why the transcripts vary in detail and the number of researchers reporting.

I have read this submission. I believe that I have an appropriate level of expertise to confirm that it is of an acceptable scientific standard.

# Response

**Published:** 2018-10-16

**Authors:** Mohammed Jawad

**Affiliations:** Imperial College London, UK

We would like thank the reviewer for their constructive comments, which we have used to improve the quality of the manuscript. The following changes have been made:

**Comment 1**: Abstract: Results: “20 representatives”, I suggest moving “20” to methods before “representatives”

**Our reply**: Thank you for this suggestion, we have moved the number of participants to the methods section of the abstract.

**Comment 2**: Para 1: Specify time for this statement“ The proportion of adolescents who have ever smoked waterpipe tobacco was between 4 and 33% in Africa and Asia, between 6 and 11% in the USA, and up to 38% in some UK 

**Our reply**: Thank you for this comment. These were taken from a systematic review which was conducted in 2008, so the prevalence estimates range from 2008 and earlier. We have since updated this review, and instead cite more recent findings with respect to prevalence. We have included the time frame in order to address the reviewer's comment. This sentence now reads:

*"A systematic review conducted in 2016 showed that the proportion of adolescents who tried waterpipe tobacco in the last 30 days averaged at 10.6% for Europe, 10.3% for the Eastern Mediterranean, and 6.8% for the Americas."*

**Comment 3**: Para 2: “about 4.1mg nicotine, 619.0mg of tar, and 192mg of carbon monoxide” remove “of”

**Our reply**: We have removed "of" from this sentence.

**Comment 4**: Para 3:“This makes designing effective interventions and policies to counter this growing trend challenging. A growing”,  I suggest replacing the first “growing” by “expanding”

**Our reply**: We have used the word "expanding" as suggested

**Comment 5**: Para 3: I suggest moving the 2014 exhibition information to the next para as it is discussed as well.

**Our reply**: Thank you for this suggestion, we have moved information about the 2014 exhibition to the next paragraph.

**Comment 6**: Written consent was not taken from the participants and this should be stated.

**Our reply**: Thank you for this comment. That written consent was not taken from the participants is stated in the Ethical Statement of the manuscript.

**Comment 7**: Since the audio recording was done following the interview, the authors should address recall bias and whether consensus between the three data collectors was necessary.

**Our reply**: Thank you for this comment. We had addressed recall bias in the limitations section of the discussion, where is it stated: *"While this could introduce recall bias, we made all efforts to record the information within minutes of the interview to maximize recall, and every interview was attended by three researchers, minimizing the possibility of bias."*

**Comment 8**: Three researchers were present at each interview but did they all participate?

**Our reply**: Yes, all researchers participated by asking questions and actively listening to the interviews. There were no predetermined allocation of roles as to whether only one person would speak, for example, in order to keep the discussions informal and natural. We have added the following sentence to the manuscript to clarify this:

*"Researchers took turns to lead each interview but all participated by asking questions and making comments in a conversational manner."*

**Comment 9**: How did you ensure privacy if others were present at the exhibition stand?

**Our reply**: Thank you for this question. It was not possible to ensure privacy during this research, given it was conducted in the public domain. We added the following sentence to include this point in our methods:

*"Privacy with participants was not possible given the public setting."*

**Comment 10**: Page 4, last para” “Rather, these manufacturers were connected with non-tobacco industries; rather than waterpipe companies reaching out to other industries..” rephrase by removing one of the two “rather”.

**Our reply**: Thank you for this comment, we have replaced the first "rather" with "instead"

**Comment 11**: Page 5, Attitude towards policy, line 4: “camping”? Correct to “ company”

**Our reply**: Thank you for spotting this typo, it has been corrected to "company".

**Comment 12**: Page 5, last para: "Our interpretation of cross-industry overlap is that waterpipe tobacco and cigarette industries are not in competition, but rather in collaboration.",  The conclusion is not supported with the results.

**Our reply**: Thank you for this comment. Having re-read the manuscript, we agree with it, and have rephrased this to:

"Our interpretation of cross-industry overlap, with particular reference to JTI's purchase of Al-Nakhla, is that waterpipe tobacco and cigarette industries are interacting with one another."

**Comment 13**: Page 6, para 1: “Furthermore, the waterpipe industry differs from tobacco in several key respects”, shouldn’t tobacco be cigarette industry?

**Our reply**: You are absolutely correct, we have replaced "tobacco" with "the cigarette industry"

# Peer review: Referee response for version 1

**DOI:** 10.5256/f1000research.14998.r33009

**Published:** 2018-04-24

**Authors:** Raed Bahelah

**Affiliations:** 1 Department of Epidemiology, Florida International University, Miami, FL, USA

This is an interesting article and well-written. I have some comments that I hope the authors find helpful:

**Introduction**

1. The context is missing in this sentence in the first paragraph: "The proportion of adolescents who have ever smoked waterpipe tobacco was between 4 and 33% in Africa and Asia, between 6 and 11% in the USA, and up to 38% in some UK communities", what is the year(s) for these estimates?
2. In the next sentence: "Waterpipe tobacco use amongst young youth in Jordan increased from 14.0% to 22.6% between 2008 and 2011, with similar patterns seen in Lebanon, Canada, and the US", do you mean ever or current waterpipe use?
3. In the second paragraph, I think the authors should add that waterpipe is perceived as less harmful and less addictive compared to cigarettes.
4. In the second paragraph, the authors need to highlight the differences between waterpipe and cigarettes as the tobacco industry techniques are well known and can be the same for waterpipe. However, one point to elaborate on, as well, is the involvement of the cigarette industry if the authors are aware of any literature, in the waterpipe business. This will support their discussion under cross-industry overlap.
5. Third paragraph, while the authors did a good job highlighting the tactics of the cigarette industry and that similar understanding is needed for the waterpipe, I did not enjoy the comparison to the alcohol industry. It is, to me, out of context here.

**Methods**

1. My main concern is that the sampling method may not necessarily capture exhibitors with "varying product type". Can the authors address this issue and justify their methodology?
2. Under "Data Collection", please make it clear that each interview was attended by all 3 researchers.
3. I am also concerned that by introducing themselves as researchers and what the study is all about, this may influence participants' responses and may intentionally lie about connection with a cigarette industry. How did the authors reduce this possible source of bias?
4. The fact that the authors audio-recorded their own discussions after each interview can by itself introduce bias. Other than recall bias, how did the authors attempted to avoid conclusion bias while discussing the interviews?
5. Following my previous question, how did the authors resolve any disagreement while transcribing the audio-recorded interviews?

**Results**

1. Some of the arguments for "Industry Growth" seem to me not really supporting growth rather globalization. For example, the proliferation of distributors and retailers in lower income countries can be due to a cheaper labor and because the waterpipe epidemic originated from the Middle East, rather than a growth. It can be also due to lax regulatory policies that allow such a proliferation. Can the authors comment on that?
2. Under "4. Attitude towards policy", please correct a typo: "For example, the owner of a major apparatus-producing camping..", should be "company" not "camping".
3. Under "4. Attitude towards policy", please correct the typo: "One example that came up.......German law from the 1970s than..", should be "that" not "than".

**Discussion**

1. The second paragraph: "Our interpretation of cross-industry overlap is that waterpipe tobacco and cigarette industries are not in competition, but rather in collaboration.", I could not see any results based on this study to support this conclusion.

**Transcripts**

1. I assume the authors reported the transcripts for 18 out of the 20 interviews. While some interviews show detailed discussions among the 3 authors, others show only "Researcher 1"?

I have read this submission. I believe that I have an appropriate level of expertise to confirm that it is of an acceptable scientific standard, however I have significant reservations, as outlined above.

# Response

**Published:** 2018-10-16

**Authors:** Mohammed Jawad

**Affiliations:** Imperial College London, UK

We would like thank the reviewer for their constructive comments, which we have used to improve the quality of the manuscript. The following changes have been made:

**Comment 1**: The context is missing in this sentence in the first paragraph: "The proportion of adolescents who have ever smoked waterpipe tobacco was between 4 and 33% in Africa and Asia, between 6 and 11% in the USA, and up to 38% in some UK communities", what is the year(s) for these estimates?

**Our reply**: Thank you for this comment, which was also raised by the other reviewer. We have added some context to this sentence and at the same time updated it a more recent systematic review of prevalence (the one originally cited was conducted in 2008). This sentence now reads:

*"A systematic review conducted in 2016 showed that the proportion of adolescents who tried waterpipe tobacco in the last 30 days averaged at 10.6% for Europe, 10.3% for the Eastern Mediterranean, and 6.8% for the Americas."*

**Comment 2**: In the next sentence: "Waterpipe tobacco use amongst young youth in Jordan increased from 14.0% to 22.6% between 2008 and 2011, with similar patterns seen in Lebanon, Canada, and the US", do you mean ever or current waterpipe use?

**Our reply**: Thank you for this comment. This refers to current (past-30 day) use, as outlined in McKelvey *et al.* 2013 (we pooled girls and boys in this calculation). We noted the reference for this trends was missing for the manuscript so we have added this in.

**Comment 3**: In the second paragraph, I think the authors should add that waterpipe is perceived as less harmful and less addictive compared to cigarettes.

**Our reply**: Thank you for this suggestion, which have now included in the first line of the manuscript and have cited a recent narrative review by Akl *et al. *to support it.

**Comment 4**: In the second paragraph, the authors need to highlight the differences between waterpipe and cigarettes as the tobacco industry techniques are well known and can be the same for waterpipe. However, one point to elaborate on, as well, is the involvement of the cigarette industry if the authors are aware of any literature, in the waterpipe business. This will support their discussion under cross-industry overlap.

**Our reply**: Thank you for this comment. The only involvement of the cigarette industry in the waterpipe industry that we are aware of is that of JTI's purchase of Nakhla tobacco, which is the predominant waterpipe tobacco brand in Egypt. This was also confirmed in our study. We are uncertain what the reviewer means by the "differences between waterpipe and cigarettes" and would welcome further clarification.

**Comment 5**: Third paragraph, while the authors did a good job highlighting the tactics of the cigarette industry and that similar understanding is needed for the waterpipe, I did not enjoy the comparison to the alcohol industry. It is, to me, out of context here.

**Our reply**: Thank you for this comment. We have removed reference to the alcohol industry.

**Comment 6**: My main concern is that the sampling method may not necessarily capture exhibitors with "varying product type". Can the authors address this issue and justify their methodology?

**Our reply**: Thank you for this question. We used a random sampling approach that factored in the size of the exhibition stand. We believe that the random component to the sampling was likely to capture exhibitions with varying product types.

**Comment 7**: Under "Data Collection", please make it clear that each interview was attended by all 3 researchers.

**Our reply**: Thank you for this comment, which was also raised by the other reviewer. We have made this clear by adding the following sentence to our methods:

*"Researchers took turns to lead each interview but all participated by asking questions and making comments in a conversational manner."*

**Comment 8**: I am also concerned that by introducing themselves as researchers and what the study is all about, this may influence participants' responses and may intentionally lie about connection with a cigarette industry. How did the authors reduce this possible source of bias?

**Our reply**: Thank you for this question. While appreciating that there is no way to be certain that our responses were completely truthful, the nature of the event was such that networking and conversations were encouraged. We did not treat our discussions as "interviews" and ensured they were kept conversational, casual and informal, while at the same time working through our interview guide. We have added the following to our limitations section:

"Finally, introducing ourselves as researchers may have influenced participants' responses. However, the nature of the event was such that networking and conversations were encouraged, and we did not expect more "truthful" answers had we not introduced ourselves as researchers, given we were unacquainted with our participants anyway." 

**Comment 9**: The fact that the authors audio-recorded their own discussions after each interview can by itself introduce bias. Other than recall bias, how did the authors attempted to avoid conclusion bias while discussing the interviews?

**Our reply**: Thank you for this question. Given how little is known about the waterpipe tobacco industry, none of the researchers had any major pre-existing beliefs about the conduct of the industry prior to these discussions. There was therefore a small possibility of conclusion (or confirmation) bias.

**Comment 10**: Following my previous question, how did the authors resolve any disagreement while transcribing the audio-recorded interviews?

**Our reply**: Thank you for this question. The transcribing and analysis were conducted by one of the team members who did not attend the exhibition. This allowed for an independent analysis of the audio content. The analyst annotated the transcripts and all three researchers responded to the annotations to clarify ambiguous statements and resolve disagreements. There were very few disagreements between the reviewers, if not none.

**Comment 11**: Some of the arguments for "Industry Growth" seem to me not really supporting growth rather globalization. For example, the proliferation of distributors and retailers in lower income countries can be due to a cheaper labor and because the waterpipe epidemic originated from the Middle East, rather than a growth. It can be also due to lax regulatory policies that allow such a proliferation. Can the authors comment on that?

**Our reply**: Thank you for this interesting point. On re-reading the transcripts we probably meant globalisation rather than growth. In fact, we could not assess actual industry growth beyond suggestive indicators, such as the fact there was an international trade fair in Germany, that there were signs of globalisation, or that products on display were quite diverse. But these could happen without industry growth. Based on this comment we have rephrased this section to refer more to globalisation than to growth.

**Comment 12**: Under "4. Attitude towards policy", please correct a typo: "For example, the owner of a major apparatus-producing camping..", should be "company" not "camping".

**Our reply**: Thank you for spotting this typo, it has been corrected.

**Comment 13**: Under "4. Attitude towards policy", please correct the typo: "One example that came up.......German law from the 1970s than..", should be "that" not "than".

**Our reply**: Thank you for spotting this typo, it has been corrected.

**Comment 14**: The second paragraph: "Our interpretation of cross-industry overlap is that waterpipe tobacco and cigarette industries are not in competition, but rather in collaboration.", I could not see any results based on this study to support this conclusion.

**Our reply: **Thank you for this comment, which was also raised by the other reviewer. Having re-read the manuscript, we agree with it, and have rephrased this to:

*"Our interpretation of cross-industry overlap, with particular reference to JTI's purchase of Al-Nakhla, is that waterpipe tobacco and cigarette industries are interacting with one another."*

**Comment 15**: I assume the authors reported the transcripts for 18 out of the 20 interviews. While some interviews show detailed discussions among the 3 authors, others show only "Researcher 1"?

**Our reply**: All researchers contributed to the discussions, but when discussions were either relatively short or straightforward, one researcher led the summary and other two only interjected in the case of disagreement or omission of content.
